# Editorial: Function and Flexibility: Friend or Foe?

**DOI:** 10.3389/fmolb.2016.00031

**Published:** 2016-07-07

**Authors:** Kris Pauwels, Peter Tompa

**Affiliations:** ^1^VIB Structural Biology Research Center, Vlaams Instituut voor BiotechnologieBrussels, Belgium; ^2^Structural Biology Brussels, Vrije Universiteit BrusselBrussels, Belgium; ^3^Research Centre for Natural Sciences of the Hungarian Academy of Sciences, Institute of EnzymologyBudapest, Hungary

**Keywords:** intrinsically disordered proteins, protein dynamics, protein flexibility, conformational ensemble, protein function

Protein structural biology aims to link snapshots of three-dimensional macromolecular structures to their biological function. The high-resolution information that is obtained traditionally by x-ray crystallography or nuclear magnetic resonance (NMR) experiments is instrumental for understanding their functional properties, their biological roles, and their potential roles in diseases (“function follows form”). Yet, proteins are not rigid and/or static entities: their dynamics and flexibility are essential for proper functioning and molecular movement, which is an important aspect of living matter. Many proteins even completely lack a well-defined 3D-structure under physiological conditions, the so-called intrinsically disordered proteins (IDPs). Up to 35% of human proteins are predicted to possess intrinsically disordered regions (IDRs) of at least 30 consecutive disordered residues, that play important roles in cell signaling and regulation (Guharoy et al., 2015) (“flexibility facilitates function”). Therefore, this research topic covers the impact of the study of protein flexibility on the structural biology field.

The articles in this e-book feature plenty of examples where protein flexibility controls protein functionality. In their fascinating *Perspective*, Kern and colleagues provide an excellent overview of our actual mechanistic insights of how the anticancer drug Gleevec selectively inhibits the Abl kinase (Agafonov et al.). Their work showcases how rigorous kinetic and structural analysis yields definitive conclusions that selectivity is a function of a conformational change after binding (induced-fit) and the resulting slow dissociation rate of Gleevec from the Abl kinase, whereby the flexibility in the famous and highly conserved DFG-loop plays an important role (Agafonov et al., 2014; Wilson et al., 2015). By reconstructing the evolution of the energy landscape of kinases through the synergy of “old-fashioned” stopped-flow kinetics and “modern” ancestral sequence reconstruction, they advocate for the combined use of experimental studies and molecular dynamics approaches to find effective and selective kinase inhibitors.

The benign role of protein flexibility is also nicely illustrated in the *review* by Gontero and colleagues who demonstrate the central and multiple functionality of C- and N-terminal intrinsically disordered tails of globular proteins in photosynthetic organisms (Thieulin-Pardo et al.). They exemplify that protein flexibility at the N- and C-terminal extremities accounts for an increased number of binding partners and how new roles may emerge by the evolutionary addition of an intrinsically disordered extension. Indeed, often IDRs play a role in molecular recognition and binding events, whereby they can undergo a folding transition induced by the partner protein (“form follows function”). By a large scale thermodynamic assessment of mostly binary protein-protein interactions of ordered-ordered and ordered-disordered protein complexes, Kragelund and colleagues help shedding light on the debated role of kinetics and thermodynamics in the binding properties of IDPs (Teilum et al.). Through this capacity for interaction with other molecules, protein flexibility can also be linked to disease (Hubin et al., 2014; Uversky, 2014; Guharoy et al., 2015). Fraternalli and colleagues studied the localization of common and disease-related mutations within (dis)ordered protein regions (Lu et al.). They highlight that intra-domain ordered and intra-domain disordered regions show high propensity for disease-related mutations, while inter-domain disordered regions are enriched in common variants. Their analysis offers interesting perspectives for the further development of the field of protein flexibility and disorder. It also supports the fact that, in the field of IDPs, computational approaches play a major role. As such, Craveur et al. show that the concept of structural alphabets is suitable to analyze the dynamics and flexibility of proteins. In their comprehensive *review* they advocate that structural alphabets are required to begin to understand the complexity of protein flexibility by discriminating flexibility from mobility and deformability.

The IDP field is also one of the few areas in structural and molecular biology where the experiments provide support to computations to achieve an accurate understanding of the conformational properties of these complex proteins. Varadi et al. review the current characterizations of IDPs by combining computations and experiments. The *mini-review* identifies key developments in the field, including the employment of experimental data into structural refinement in search of the functional repertoire of IDPs. With regard to wet-lab experimental approaches, several emerging techniques allow to overcome some of the technical problems of studying IDPs and to obtain essential information on protein dynamics. In their *original research paper*, Barran and collaborators exemplify the potential of ion-mobility mass spectrometry to track conformational changes in unstructured proteins on a millisecond timescale (Dickinson et al.). They characterize the effect of two small molecule compounds RITA and nutlin-3 on their IDP targets with a multi-technique approach. The *minireview* by Belle and coworkers showcases the power of site-directed spin labeling with electron paramagnetic resonance to investigate flexible regions and fuzziness in proteins (Le Breton et al.). The information obtained by NMR can generate conformational ensembles that visualize the conformations that IDPs sample under functional conditions. Because protein disorder can be evaluated at the residue level with NMR, Nielsen and Mulder compiled a small database of disorder-containing proteins using experimental NMR chemical shift data in their *original research paper* that is felicitously entitled “There is Diversity in Disorder – ‘In all Chaos there is a Cosmos, in all Disorder a Secret Order”’. They demonstrate that those proteins span the full spectrum of disorder, yet segregate into two classes: proteins mostly disordered but with small segments of order scattered along the sequence, or structured proteins with small segments of disorder inserted between the different structured regions. This study is also illustrative for the concept of “form and function follow (NMR) frequency.”

Recently the D^3^-concept was introduced for IDPs by revealing the interconnections between protein intrinsic Disorder and Degenerative Diseases (Uversky, 2014). In analogy, it is opportune to introduce the F^3^-concept for flexible proteins, since “Function Follows Flexibility.” Whereas in the past intrinsic disorder could cause frustration because IDRs were considered frivolous and flamboyant, their flirtatious behavior flaunted formidable features. We hope this e-book can stimulate the research community to finally stop fumbling for the fugacious forms of flexible proteins and bring their functional framing to fruition.

## Author contributions

Both authors made substantial, direct and intellectual contribution to the work, and approved it for publication.

### Conflict of interest statement

The authors declare that the research was conducted in the absence of any commercial or financial relationships that could be construed as a potential conflict of interest.
